# Incidental Thrombus in Transit Causing Embolic Stroke

**DOI:** 10.1155/2017/3161972

**Published:** 2017-06-20

**Authors:** Brian Malm, Paul Hermany, Alan Morrison

**Affiliations:** ^1^Section of Cardiovascular Medicine, Department of Internal Medicine, Yale University School of Medicine, Yale University, New Haven, CT, USA; ^2^Department of Cardiology, VA Connecticut Healthcare System, West Haven, CT, USA; ^3^Warren Alpert School of Medicine, Brown University, Providence, RI, USA

## Abstract

Thrombus in transit leading to paradoxical systemic arterial embolism is a rare echocardiographic finding in patients presenting with embolic stroke. We present a case of a patient who had an atrial thrombus in transit discovered incidentally and later suffered a fatal stroke. Etiologies of cardioembolic stroke and the use of echocardiography in diagnosis and management are briefly discussed.

## 1. Case Presentation

**Figure 1:**
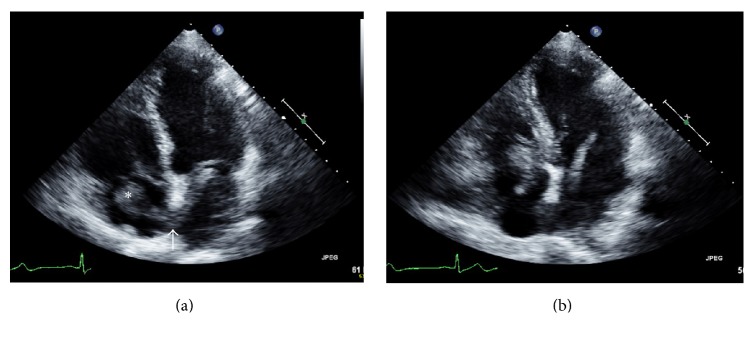
(a) Transthoracic echocardiogram apical four-chamber view in diastole showing a large thrombus (asterisk) extending from the right atrium to the left atrium via a patent foramen ovale (arrow). (b) Apical four-chamber view in diastole demonstrating prolapse of the thrombus across the mitral and tricuspid valves.

A 68-year-old male with Parkinson's disease was referred for an echocardiogram by his primary care physician as part of an outpatient work-up for intermittent hypotension and bradycardia. He denied any other cardiovascular or transient neurological symptoms. He uses a walker for ambulation. His past medical history is also notable for hypertension, hyperlipidemia, and osteoarthritis. He has had no prior cardiac testing. His current medications include alprostadil, amantadine, carbidopa/levodopa, entacapone, loratadine, midodrine, oxybutynin, ropinirole, and simvastatin. He also receives botulin toxin injections for muscular rigidity. He is an ex-smoker and drinks alcohol in moderation. His family history is notable only for hypertension. He previously worked as an accountant. His physical exam was notable for slow gait and resting tremor and was otherwise unremarkable including normal blood pressure. His transthoracic echocardiogram demonstrated a large highly mobile elongated mass consistent with thrombus (asterisk Figure 1(a)) in the right atrium which crosses the interatrial septum, presumably through a patent foramen ovale (arrow Figure 1(a)) into the left atrium. The thrombus also prolapses across both the tricuspid and mitral valves during diastole (Figure 1(b)) and measures greater than 10 cm in length. The patient was sent to the nearest emergency department, started on heparin and admitted to the hospital for further management. Shortly after admission the patient suffered a massive and ultimately fatal, left middle cerebral artery stroke.

## 2. Case Discussion

Cerebrovascular events are a leading cause of morbidity and mortality in the United States and the majority of these are ischemic in etiology [1]. Cardioembolic etiology of ischemic stroke accounts for approximately 20% of all cases and occurs most often in the setting of high risk conditions including, but not limited to, atrial fibrillation, intracardiac mass/thrombus, rheumatic mitral valve disease, prosthetic heart valves, complex aortic atheroma, and dilated cardiomyopathies [2, 3]. In patients with cryptogenic stroke where a causal cardioembolic source has not been identified on routine evaluation, there is an increased incidence of patent foramen ovale (PFO) implicating paradoxical embolization as a potential causative mechanism for stroke in these patients, though this is often challenging to demonstrate, especially considering the prevalence of PFO is >25% of the population [4, 5]. Treatment options for patients with cardioembolic stroke in the setting of a PFO include antithrombotic therapy and percutaneous or surgical closure. Selection of appropriate candidates for, and the utility of, percutaneous PFO closure remains uncertain given conflicting evidence for benefit from randomized clinical trials [6, 7], though recently presented 10-year results from the RESPECT trial demonstrate significant stroke reduction with PFO closure compared to medical therapy alone [8]. This finding led to subsequent FDA approval of the Amplatzer PFO occluder device.

Diagnostic imaging remains the cornerstone for accurate diagnosis and risk stratification in patients with cerebrovascular disease and stroke. Echocardiography is widely available and remains the current standard imaging modality for the evaluation of stroke patients with a suspected cardioembolic source. Use of transthoracic echocardiography with harmonic imaging and agitated saline contrast allows for the accurate diagnosis of suspected right-to-left shunt through a PFO/ASD in patients diagnosed with cardioembolic stroke [9]. In selected cases, transesophageal echocardiography is necessary for diagnosis of interatrial shunt and accurate characterization of the atrial septal anatomy, particularly when device closure is being considered [10]. In addition, atrial septal aneurysms have been associated with cryptogenic stroke and right-to-left shunting and may be missed on TTE [11, 12].

Thrombus in transit leading to systemic arterial embolism is a rare echocardiographic finding in patients presenting with embolic stroke. These thrombi typically originate from the deep veins of the pelvis and proximal lower extremities but may also be seen in patients with certain malignancies such as renal cell carcinoma. Arterial embolization in this setting requires communication between the right and left heart, most commonly through a PFO. Paradoxical embolization is most often reported in the setting of pulmonary embolism which may lead to acutely increased right heart pressures favoring right-to-left shunting through a PFO [13]. Reports of thrombus in transit in the literature are most often described in patients undergoing diagnostic testing in the setting of a stroke [14]. This case is unique as the interatrial thrombus in transit was discovered incidentally on transthoracic echocardiography ordered for an unrelated indication. Management options include anticoagulation, inferior vena cava filter placement, and prompt consideration for surgical removal. Despite these interventions, the reported 30-day mortality of this finding approaches 20% [13].

## Supplementary Material

Transthoracic echocardiogram apical four-chamber view showing a large highly mobile thrombus extending from the right atrium to the left atrium via a patent foramen ovale. There is also prolapse of the thrombus across the mitral and tricuspid valves during diastole.
